# The Impact of Repeated Applications of Botulinum Toxin A on the Spasticity of the Upper Limb in Stroke Patient, Cohort Study

**DOI:** 10.3390/jcm14082735

**Published:** 2025-04-16

**Authors:** Nilüfer Aygün Bilecik, Serpil Tuna, Özlem Karataş, Nilüfer Balcı

**Affiliations:** 1 Physical Medicine and Rehabilitation, Adana City Hospital, Health Sciences University, 01230 Adana, Turkey; 2 Physical Medicine and Rehabilitation, Faculty of Medicine, Akdeniz University, 07058 Antalya, Turkey; serpiltuna@akdeniz.edu.tr (S.T.); drozlemkaratas86@gmail.com (Ö.K.); nilbalci@akdeniz.edu.tr (N.B.)

**Keywords:** spasticity, stroke patients, Botulinum Toxin A, Modified Ashworth Scores, Brunnstrom stage

## Abstract

**Background:** Spasticity is a muscle stiffness issue often caused by spinal cord or cerebral diseases, notably impairing stroke patients. This study aims to evaluate the long-term effectiveness of repeated Botulinum Toxin A injections on spasticity and arm function, to understand if the treatment’s effects accumulate or diminish over time. **Methods:** This study examines 85 stroke patients treated with one or five sessions of BTX-A injections between 2013 and 2019. Patients were divided into two groups based on the number of sessions and evaluated using Modified Ashworth Scores, Brunnstrom Stage, and Frenchay Arm Test. **Results:** This study includes 85 hemiplegic patients with an average age of around 50, and various muscle groups were treated with BTX-A injections. Group 2, who received five sessions, showed significantly greater improvement in MAS scores for certain muscle groups and had higher FAT scores compared to Group 1, who received just one session. Overall, BTX-A treatment led to significant improvements in MAS, Brunnstrom, and FAT scores across all patients. **Conclusions:** Our findings corroborate existing literature by affirming that Botox injections effectively mitigate spasticity and enhance arm functionality. Notably, our data reveal that repeated Botox treatments yield significantly greater benefits in reducing spasticity in the elbow pronators, wrists, and finger flexors compared to a single session. This study uniquely demonstrates the cumulative benefits of multiple BTX-A sessions, highlighting that repeated applications not only sustain but also amplify functional improvements over time. These results advocate for the feasibility and augmented effectiveness of administering a series of five Botox injections in the management of post-stroke spasticity.

## 1. Introduction

Spasticity is a motor control disorder characterized by heightened muscle tone and resistance to passive movement, commonly resulting from neurological conditions affecting the spinal cord or brain, leading to significant disability in affected individuals [1].

It is a major contributor to morbidity and disability in stroke patients, impairing their quality of life and daily functional abilities while also increasing the risk of complications [2]. Various therapeutic approaches are employed to manage spasticity, including physical therapy, oral antispasmodics, neuromuscular blocks, intrathecal baclofen, and surgical interventions. Among these, Botulinum Toxin A (BTX-A) has emerged as the preferred pharmacological option for focal spasticity management due to its prolonged but reversible effects, safety, and minimal side effect profile [3,4,5]. The therapeutic effects of BTX-A typically last for 2 to 3 months, after which nerve transmission resumes through axonal sprouting and the formation of new synaptic junctions [1]. Consequently, repeated injections are often necessary to maintain therapeutic efficacy. However, there is limited research examining the long-term effects of repetitive BTX-A administration on spasticity and its associated complications [6,7,8]. Furthermore, the literature presents conflicting findings regarding the cumulative benefits and potential adverse effects of multiple BTX-A applications. To our knowledge, no study has comprehensively assessed the impact of repeated BTX-A injections on arm functionality and Brunnstrom staging in stroke patients.

The aim of this study is to evaluate the efficacy of repeated BTX-A injections on spasticity and upper limb functionality in stroke patients. Specifically, we seek to compare the outcomes of repeated sessions with those of the initial session to determine whether therapeutic effects accumulate or diminish with successive applications.

## 2. Materials and Methods

This retrospective study evaluated 85 stroke patients who received either one or five sessions of BTX-A injections between 2013 and 2019 at Akdeniz University. Ethical approval for this study was obtained from the Clinical Research Ethics Committee of Akdeniz University Faculty of Medicine (Approval date: 29 November 2020, Reference number: 70904504/794).

Physical Medicine and Rehabilitation Clinic. A standardized BTX-A preparation (Botox^®^, 100 U) diluted with 2 mL of saline was administered at 3-month intervals. Treatment efficacy was assessed one month after each injection using Modified Ashworth Scores (MAS), Brunnstrom Staging, and the Frenchay Arm Test (FAT) obtained from patient records. MAS was used to evaluate spasticity, Brunnstrom Staging was used for neurophysiological function, and FAT was used for arm functionality.

This study included patients diagnosed with ischemic stroke who had developed upper limb spasticity. Only patients in the chronic phase of stroke (≥6 months post-stroke) were included to ensure that treatment effects were evaluated in a stable neurological state. Stroke severity criteria required that all included patients had moderate-to-severe upper limb spasticity (Modified Ashworth Scale ≥ 2). Additionally, patients were required to have residual voluntary movement in the affected upper limb to allow functional assessment using Brunnstrom Staging and the Frenchay Arm Test (FAT). The following exclusion criteria were applied: patients with hemorrhagic stroke; patients who had received prior BTX-A treatment before enrollment in this study; patients with severe joint contractures that would prevent functional assessment; patients with cognitive impairments or communication difficulties that could interfere with accurate outcome evaluations.

All Modified Ashworth Scale (MAS), Brunnstrom Staging, and Frenchay Arm Test (FAT) assessments were conducted by a single, experienced physical medicine and rehabilitation specialist. This assessor was trained in the standardized administration of these scales to ensure consistency and reliability in the measurements.

Patients were classified into two groups. Group 1 consisted of those receiving a single session (66 patients), and Group 2 included those undergoing five sessions. The groups were compared based on initial, pre-session, and post-session MAS, Brunnstrom, and FAT scores. Additionally, changes from initial to post-session values were analyzed to assess the cumulative effect of BTX-A treatment.

All statistical analyses were performed using SPSS 21 software. Given the non-normal distribution of MAS, Brunnstrom, and FAT values, non-parametric tests were employed. Between-group differences were evaluated using the Mann–Whitney U test, while intra-group comparisons were made using the Friedman test and Wilcoxon Signed Rank test for pairwise comparisons. Bonferroni correction was applied, with a significance level set at *p* < 0.05.

## 3. Results

The average age of the total 85 hemiplegic patients (52 males, 33 females) was found to be 49.89 ± 15.18 (18–88). The demographic and clinical characteristics of the patients are given in Table 1.

Out of the total 85 patients, BTX-A was applied to the elbow flexors in 69, the pronators in 62, the wrist flexors in 72, and the finger flexors in 74. When the MAS scores recorded at the initial examination of the groups were compared, the finger flexor MAS values of Group 2 were found to be significantly higher than those of Group 1 (*p* = 0.021). When pre-session and post-session MAS scores were compared between the groups, the pronator and wrist flexor MAS values of Group 2 were significantly lower than those of Group 1 (*p* < 0.05). (Table 2) Additionally, the difference between post-session and initial examination MAS scores for the pronators, wrist, and finger flexors was significantly greater in Group 2 compared to Group 1. (*p* < 0.05) (Table 2) Pre-session and post-session FAT scores were also significantly higher in Group 2 compared to Group 1. (*p* < 0.05). No differences were found in the Brunnstrom staging scores between the groups for initial examination, pre-session, and post-session values, but when comparing the difference between post-session and initial examination, there was a significantly greater increase in Brunnstrom arm scores in Group 2 (*p* = 0.001) (Table 2).

When comparing all patients’ initial examination, pre-session, and post-session values for Botox effectiveness, a significant decrease in all MAS scores (*p* < 0.05), a significant increase in Brunnstrom hand stage (*p* = 0.014), and a significant increase in FAT scores (*p* = 0.005) were observed (Table 3).

## 4. Discussion

In this study, we evaluated the impact of repeated BTX-A injections administered at regular intervals on upper limb spasticity in hemiplegic patients. Both single-session and fifth-session BTX-A injections significantly reduced spasticity and improved arm function. However, five sessions of BTX-A were found to be more effective in reducing spasticity in the elbow pronators, wrist, and finger flexors compared to a single session. Additionally, multiple sessions provided greater improvements in overall arm function.

Spasticity is a motor control disorder resulting from various pathologies affecting the spinal cord or brain, often leading to significant disability. Although the underlying pathophysiological mechanisms of spasticity are not yet fully elucidated, it is generally associated with increased motor neuron excitability due to impaired lower motor neuron inhibition and predominant upper motor neuron activity [1,2]. In stroke patients, spasticity, seen in 20–40% of cases, is one of the main causes of morbidity and disability [3].

Botulinum toxin, produced by the bacterium *Clostridium botulinum*, induces a reversible blockade at the neuromuscular junction. There are eight identified serotypes of botulinum toxin (A, B, C1, C2, D, E, F, and G) [1]. Serotype A, first utilized by Das and Park in 1989 for stroke-induced spasticity, has since become the preferred pharmacological treatment for focal spasticity due to its prolonged yet reversible effect, reliability, and favorable safety profile [3,4,5]. The Modified Ashworth Scale (MAS) is the most widely used method for assessing spasticity severity [8]. Prior studies have demonstrated significant reductions in MAS scores following BTX-A treatment [8,9,10]. In our study, although improvements were observed after the initial session, patients who received five sessions exhibited significantly greater reductions in spasticity compared to their initial evaluations.

Botulinum toxin (BTX-A) injections have proven to be an effective therapeutic option for managing spasticity associated with various neurological conditions, including stroke, multiple sclerosis, brain injuries, spinal cord injuries, and cerebral palsy. Compared to oral antispastic agents, BTX-A offers distinct advantages due to its localized action and minimal systemic side effects. Several studies have validated the efficacy and safety of BTX-A in reducing spasticity, particularly in the upper extremities of hemiplegic patients [11,12]. Brashear et al. [11] reported a reduction in spasticity and disability in the wrist and finger flexors in 126 hemiplegic patients treated with Botox in a multi-center, placebo-controlled study. Wang et al. [9] reported, in a study with 16 patients, that Botox treatment significantly reduced muscle tone and pain, but there was no significant improvement in functional status. The discrepancy between our findings and those of Wang et al. [9], who did not observe functional improvements following BTX-A treatment, can be attributed to several factors. Firstly, Wang et al.’s study had a small sample size of only 16 patients, which may limit the generalizability and statistical power of their results. In contrast, our study included 85 patients, providing a more robust dataset for detecting functional changes. Secondly, differences in treatment protocols, such as the dosage of BTX-A and the frequency of injections, may have influenced the outcomes. Wang et al. may have used a lower dose or less frequent administration, which could result in less pronounced functional gains. Additionally, the assessment tools and timing of evaluations might have varied between the studies. Our study employed the Frenchay Arm Test (FAT) to measure arm functionality, which may be more sensitive in detecting subtle improvements compared to the functional assessments used by Wang et al. Furthermore, patient characteristics, including the severity of spasticity and the stage of stroke recovery, could differ between the two studies, potentially affecting the responsiveness to BTX-A treatment. Lastly, the longer follow-up period in our study allowed for the observation of sustained functional improvements, whereas Wang et al.’s study may have had a shorter follow-up duration, limiting the ability to detect long-term benefits. These factors collectively may explain why our study observed significant functional improvements while Wang et al. did not. In our study, Botox injection applied to the upper extremity in hemiplegic patients was also found to be effective on spasticity in both the hand and arm.

Despite the overall efficacy of repeated BTX-A injections in reducing spasticity and improving arm function, certain muscle groups, such as the finger flexors, did not exhibit the same level of improvement. Several factors may contribute to this differential response. Firstly, the anatomical and functional characteristics of the finger flexors differ significantly from larger muscle groups like the elbow pronators and wrist flexors. Finger flexors are smaller and more intricate, making precise localization and adequate dosing of BTX-A more challenging [13]. Insufficient toxin distribution within these smaller muscles may result in less pronounced therapeutic effects. Secondly, the baseline severity and pattern of spasticity in finger flexors may vary, potentially requiring higher or more frequent doses to achieve similar outcomes [14]. Additionally, the finger flexors are involved in fine motor movements, and even minor residual spasticity can significantly impact functional improvements, making measurable gains less apparent [15]. Another consideration is the variability in assessment sensitivity; the tools and scales used to evaluate spasticity and functionality in finger flexors may not be as sensitive or specific as those used for larger muscle groups, potentially underestimating the actual improvements [15,16]. Furthermore, compensatory mechanisms may play a role, where the reduction of spasticity in larger muscle groups leads to increased reliance on finger flexors, thereby limiting their functional recovery [17]. Lastly, patient-specific factors such as adherence to rehabilitation protocols, individual variability in response to BTX-A, and the presence of concurrent therapies can influence the degree of improvement observed in different muscle groups [18]. Future studies should explore optimized dosing strategies, targeted injection techniques, and more sensitive assessment tools to enhance the therapeutic outcomes for finger flexors in hemiplegic patients.

The cumulative therapeutic benefits observed with repeated BTX-A injections may be attributed to several underlying biological mechanisms, including neural plasticity and synaptic recovery. Neural plasticity refers to the brain’s ability to reorganize itself by forming new neural connections, which can be enhanced through a consistent reduction of spasticity and subsequent motor rehabilitation [19]. Repeated BTX-A administration leads to sustained muscle relaxation, which not only alleviates spasticity but also facilitates motor learning and the reestablishment of normal movement patterns [20]. This sustained reduction in abnormal muscle tone may promote synaptic recovery and the normalization of neurotransmitter release at the neuromuscular junction, thereby enhancing motor neuron function over time [21]. Additionally, BTX-A has been shown to modulate sensory feedback from muscles, which can further contribute to cortical reorganization and improved motor control [22]. These neurophysiological changes collectively support the notion that repeated BTX-A injections can induce long-term improvements in motor function by fostering an environment conducive to neural and synaptic rehabilitation. Future research should aim to elucidate these mechanisms in greater detail to optimize treatment protocols and maximize therapeutic outcomes for hemiplegic patients.

The findings of our study have significant implications for current treatment protocols and patient counseling practices in managing upper limb spasticity in hemiplegic patients. Our observation that repeated BTX-A injections provide cumulative therapeutic benefits suggests that adopting a structured injection schedule with regular intervals can enhance treatment efficacy. Clinicians may consider extending the treatment regimen beyond a single session to achieve more sustained and comprehensive reductions in spasticity, particularly in key muscle groups such as the elbow pronators and wrist flexors. Additionally, our study underscores the importance of personalized treatment plans, where injection frequency and dosage are tailored to the individual patient’s response and specific muscle involvement.

In terms of patient counseling, these findings allow healthcare providers to set more accurate expectations regarding the treatment course and potential outcomes. Patients can be informed that while initial improvements may be observed after the first injection, optimal functional gains are more likely with continued therapy over multiple sessions. This information can enhance patient adherence to the treatment plan and improve overall satisfaction with the therapy. Furthermore, emphasizing the favorable safety profile and minimal adverse effects observed in our study can alleviate patient concerns and encourage the acceptance of repeated BTX-A injections as a viable long-term management strategy for upper limb spasticity. Future guidelines should incorporate these insights to refine treatment protocols and optimize patient-centered care in spasticity management.

The literature presents conflicting evidence on whether repeated BTX-A injections lead to cumulative benefits or diminished effects due to antibody development over time. Bakheit et al. [23] suggested that the positive effects of BTX-A persisted after at least three sessions. Similarly, Elovic et al. [7] reported that five sessions were well-tolerated and significantly reduced muscle tone in post-stroke spasticity. Catz et al. [24] noted significant improvements following a single session, with further gains observed in subsequent sessions. Çoban et al. [8] found that MAS scores were significantly lower in the forearm pronator muscles after the fifth injection compared to the first. Repeated BTX-A injections have been shown to provide cumulative therapeutic benefits in managing spasticity. Bakheit et al. [23] demonstrated that the positive effects of BTX-A persisted after at least three sessions, while Elovic et al. [7] reported significant reductions in muscle tone with five repeated injections. Çoban et al. [8] found sustained improvements in forearm pronator muscles following multiple BTX-A treatments. Additionally, Fattal-Valevski et al. [25] studied the long-term effects of repeated BTX-A injections in children with cerebral palsy and showed that they provided permanent improvements in gross motor function. These studies collectively reinforce the notion that repeated BTX-A applications can enhance therapeutic outcomes and offer cumulative benefits in hemiplegic patients. Consistent with these findings, our study demonstrated that MAS scores in the elbow flexors, pronator muscles, and wrist flexors significantly decreased after five sessions compared to a single session. However, this effect was not observed in the elbow extensors and finger flexors. The increased efficacy in repeated applications could be due to the reapplication of Botox before its full effect has worn off in the muscle. Another view is that patients are adapting to the reduced muscle tone after the injections, and they are showing better performance in functional tests [8].

In this study, we also assessed the functional status of the upper extremities, and a significant improvement was observed in all patients following BTX-A administration. Similarly, Atalay et al. [26] reported in their study that Botox injection was effective on arm functions, aligning with the findings of our study.

During the treatment process, no significant adverse effects or tolerability issues were observed in any of the patients receiving BTX-A injections. The injections were well-tolerated, and patients did not report any severe side effects such as muscle weakness, dysphagia, or allergic reactions. These findings are consistent with previous studies that have demonstrated the favorable safety profile of BTX-A in managing spasticity [27,28,29]. The absence of notable adverse effects in our study supports the use of repeated BTX-A injections as a safe and effective treatment option for upper limb spasticity in hemiplegic patients. However, it is important to monitor patients for potential side effects in future studies, especially in larger and more diverse populations.

This study has several limitations that should be acknowledged. Firstly, the retrospective design inherently introduces potential biases, such as selection bias, which may affect the generalizability of the findings. The reliance on patient records limits the ability to control for all confounding variables, potentially impacting the accuracy of the results. Additionally, this study was conducted at a single center with a relatively small sample size of 85 patients, which may not fully represent the broader population of hemiplegic stroke patients. The absence of a control group receiving standard care without BTX-A injections further restricts the ability to attribute improvements solely to the treatment. Future prospective, multi-center studies with larger cohorts are necessary to validate these findings and to explore the long-term effects of repeated BTX-A injections on upper limb spasticity and functionality in hemiplegic patients. Additionally, all MAS, Brunnstrom Staging, and FAT assessments were conducted by a single assessor, and inter-rater reliability was not evaluated. This could introduce measurement bias and limit the objectivity of the assessment scores. Future studies should consider involving multiple assessors and evaluating inter-rater reliability to enhance the robustness of the findings.

Future research should focus on determining the optimal interval between BTX-A injections to maximize therapeutic benefits while minimizing potential side effects. Additionally, exploring different BTX-A formulations could provide insights into enhancing treatment efficacy and patient outcomes. Investigating these aspects will help in refining treatment protocols and personalizing therapy for hemiplegic patients with upper limb spasticity.

Despite these limitations, our study aligns with the literature, demonstrating that BTX-A reduces spasticity and improves arm function. Moreover, repeated BTX-A sessions yielded greater improvements in spasticity of the elbow pronators, wrist, and finger flexors compared to a single session. These results suggest that a series of five BTX-A injections is both well-tolerated and effective in managing post-stroke spasticity, potentially offering cumulative therapeutic benefits.

## Figures and Tables

**Table 1:** Demographic and clinical characteristics of the patients.

		Group 1	Group 5	*p*-Value	Total
		(50)	(35)		(85)
Gender	Female n (%)	21 (42%)	12 (34%)	0.506	33 (39%)
	Male n (%)	29 (58%)	23 (66%)		52 (61%)
Age (mean ± std)		50.96 ± 2.16	48.37 ± 2.56	0.442	49.89 ± 15.18
Stroke year duration (median (min-max))		5 (1–18)	3 (1–12)	0.266	4 (1–18)
Etiology	Ischemic	33 (66%)	16 (46%)		49 (58%)
	Hemorrhagic	12 (24%)	18 (51%)	0.025 *	30 (35%)
	Other	5 (10%)	1 (3%)		6 (7%)
Affected side	Right	28 (61%)	18 (39%)	0.825	46 (54%)
	Left	22 (56%)	17 (44%)		39 (46%)

*: *p* < 0.05; Group 1: Patients who received 1st session Botox treatment; Group 2: Patients who received 5th session Botox treatment.

**Table 2:** Comparison of MAS, Brunstom staging, and FAT values of patients who underwent one or five sessions of Botox.

			Group 1 (50) Median (Q1–Q3)	Group 5 (35) Median (Q1–Q3)	*p*-Value
**MAS**	Elbow flexors	İnitial examination	2(2–3)	3(2–3)	0.362
		Pre-session	2(2–3)	2(2–2)	0.127
		Post-session	2(1–2)	2(1–2)	0.285
		Post-initial exam different	−1(−1–0)	−1(−1–0)	0.067
	Forearm pronators	İnitial examination	2(2–3)	3(2–3)	0.164
		Pre-session	2(2–3)	2(2–2)	0.023 *
		Post-session	2(1–2)	1(1–2)	0.027 *
		Post-initial exam different	−0.5(−1–0)	−1(−2–−1)	0.001 *
	Wrist flexors	İnitial examination	2(2–3)	3(2–3)	0.314
		Pre-session	2(2–3)	2(2–2)	0.024 *
		Post-session	2(2–2)	2(1–2)	0.019 *
		Post-İnitial exam different	−0.5(−1–0)	−1(−1–0)	0.002 *
	Finger flexors	İnitial examination	3(2–3)	3(3–3)	0.021 *
		Pre-session	3(2–3)	2(2–3)	0.147
		Post-session	2(2–3)	2(2–2)	0.232
		Post-initial exam different	−0.5(−1–0)	−1(−1–−1)	0.001 *
**BR**	Arm	İnitial examination	3(2–3)	3(2–3)	0.459
		Pre-session	3(2–3)	3(3–3)	0.188
		Post-session	3(2–3)	3(3–3)	0.091
		Post-initial exam different	0(0–0)	0(0–1)	0.001 *
	Hand	İnitial examination	2(2–3)	2(1–3)	0.359
		Pre-session	2(2–3)	2(2–3)	0.395
		Post-session	2(2–3)	2(2–3)	0.483
		Post-initial exam different	0(0–0)	0(0–0)	0.260
**FAT**		İnitial examination	İnitial examination	1(0–2)	0.797
		Pre-session	Pre-session	2(1–2)	0.017 *
		2	Post-session	2(1–2)	0.025 *
		2–0	Post-initial exam different	0(0–1)	0.006 *

*: *p* < 0.05. MAS: Modified Ashworth Scale, BR: Brunnstrom Stage, FAT: The Frenchay Arm Test, 0: İnitial examination, 1: Pre-session, 2: Post-session, 2–0: Post-session—İnitial examination difference. Group 1: Patients who received a single session of Botox. Group 2: Patients who received five sessions of Botox.

**Table 3:** Effect of Botox treatment on MAS, Brunstom stage, and FAT values in all patients.

		0 (İnitial Examination) Median (Q1–Q3)	1 (Pre-Session) Median (Q1–Q3)	2 (Post-Session) Median (Q1–Q3)	*p*	*p* (0–1)	*p* (0–2)	*p* (1–2)
**MAS**	Elbow flexors	2(2–3)	2(2–3)	2(1–2)	<0.01 *	<0.01 **	<0.01 **	<0.01 **
	Forearm pronators	2(2–3)	2(2–3)	2(1–2)	<0.01 *	<0.01 **	<0.01 **	<0.01 **
	Wrist flexors	2(2–3)	2(2–3)	2(1–2)	<0.01 *	<0.01 **	<0.01 **	<0.01 **
	Finger flexors	3(2–3)	3(2–3)	2(2–3)	<0.01 *	<0.01 **	<0.01 **	<0.01 **
**BR**	Arm	3(2–3)	3(2–3)	3(2–3)	<0.01 *	<0.01 **	<0.01 **	0.655
	Hand	2(2–3)	2(2–3)	2(2–3)	0.115	0.157	0.096	0.317
**FAT**		1(0–2)	1(1–2)	1(1–2)	<0.01 *	0.001 **	0.001 **	0.025

*: *p* < 0.05, **: *p* < 0.017 (Bonferroni correction), MAS: Modified Ashworth Scale, BR: Brunnstrom Stage, FAT: The Frenchay Arm Test.

## Data Availability

The original contributions presented in this study are included in the article. Further inquiries can be directed to the corresponding author.

## References

[1] Karaçam M., Selçuki D. (2010). The efficacy of botulinum toxin A intramuscular injections in after-stroke spasticity. Turk. J. Neurol..

[2] Yablon S.A., Brashear A., Gordon M.F., Elovic E.P., Turkel C.C., Daggett S., Liu J., Brin M.F. (2007). Formation of neutralizing antibodies in patients receiving botulinum toxin type A for treatment of poststroke spasticity: A pooled-data analysis of three clinical trials. Clin. Ther..

[3] Das T., Park D. (1989). Effect of treatment with botulinum toxin on spasticity. Postgrad. Med. J..

[4] Das T., Park D. (1989). Botulinum toxin in treating spasticity. Br. J. Clin. Pract..

[5] Farmer S.F., Harrison L.M., Ingram D.A., Stephens J.A. (1991). Plasticity of central motor pathways in children with hemiplegic cerebral palsy. Neurology.

[6] Chang M.A. (2015). Possible adverse effects of repeated botulinum toxin A injections to decrease post-stroke spasticity in adults undergoing rehabilitation: A review of the literature. J. Allied Health.

[7] Elovic E.P., Brashear A., Kaelin D., Liu J., Millis S.R., Barron R., Turkel C. (2008). Repeated treatments with botulinum toxin type a produce sustained decreases in the limitations associated with focal upper-limb poststroke spasticity for caregivers and patients. Arch. Phys. Med. Rehabil..

[8] Coban A., Matur Z., Hanagasi H.A., Parman Y. (2014). Efficiency of repeated botulinum toxin type-A injections in post-stroke distal limb spasticity. J. Neurol. Sci. Turk..

[9] Wang H.C., Hsieh L.F., Chi W.C., Lou S.M. (2002). Effect of intramuscular botulinum toxin injection on upper limb spasticity in stroke patients. Am. J. Phys. Med. Rehabil..

[10] Koçak F.A., Aras M., Köseoğlu B.F. (2014). İnmeli hastalarda üst ekstremite spastisitesinin botulinum toksin tip A ile tedavisi. J. Phys. Med. Rehabil. Sci..

[11] Brashear A., Gordon M.F., Elovic E., Kassicieh V.D., Marciniak C., Do M., Lee C.H., Jenkins S., Turkel C. (2002). Intramuscular injection of botulinum toxin for the treatment of wrist and finger spasticity after a stroke. N. Engl. J. Med..

[12] Cardoso E., Rodrigues B., Lucena R., Oliveira I.R., Pedreira G., Melo A. (2005). Botulinum toxin type A for the treatment of the upper limb spasticity after stroke: A meta-analysis. Arq. Neuropsiquiatr..

[13] Picelli A., Roncari L., Baldessarelli S., Berto G., Lobba D., Santamato A., Fiore P., Smania N. (2014). Accuracy of botulinum toxin type A injection into the forearm muscles of chronic stroke patients with spastic flexed wrist and clenched fist: Manual needle placement evaluated using ultrasonography. J. Rehabil. Med..

[14] Santamato A. (2022). High Doses of Botulinum Toxin Type A for the Treatment of Post-Stroke Spasticity: Rationale for a Real Benefit for the Patients. Toxins.

[15] Plantin J., Pennati G.V., Roca P., Baron J.C., Laurencikas E., Weber K., Godbolt A.K., Borg J., Lindberg P.G. (2019). Quantitative Assessment of Hand Spasticity After Stroke: Imaging Correlates and Impact on Motor Recovery. Front. Neurol..

[16] Guo X., Wallace R., Tan Y., Oetomo D., Klaic M., Crocher V. (2022). Technology-assisted assessment of spasticity: A systematic review. J. Neuroeng. Rehabil..

[17] Cirstea M.C., Levin M.F. (2000). Compensatory strategies for reaching in stroke. Brain.

[18] Lee J.I., Jansen A., Samadzadeh S., Kahlen U., Moll M., Ringelstein M., Soncin G., Bigalke H., Aktas O., Moldovan A.S. (2021). Long-term adherence and response to botulinum toxin in different indications. Ann. Clin. Transl. Neurol..

[19] Rogozhin A.A., Pang K.K., Bukharaeva E., Young C., Slater C.R. (2008). Recovery of mouse neuromuscular junctions from single and repeated injections of botulinum neurotoxin A. J. Physiol..

[20] Yu H.X., Liu S.H., Wang Z.X., Liu C.B., Dai P., Zang D.W. (2022). Efficacy on gait and posture control after botulinum toxin A injection for lower-limb spasticity treatment after stroke: A randomized controlled trial. Front. Neurosci..

[21] Machamer J.B., Vazquez-Cintron E.J., Stenslik M.J., Pagarigan K.T., Bradford A.B., Ondeck C.A., McNutt P.M. (2023). Neuromuscular recovery from botulism involves multiple forms of compensatory plasticity. Front. Cell Neurosci..

[22] Veverka T., Hlustik P., Hok P., Otruba P., Tudos Z., Zapletalova J., Krobot A., Kanovsky P. (2014). Cortical activity modulation by botulinum toxin type A in patients with post-stroke arm spasticity: Real and imagined hand movement. J. Neurol. Sci..

[23] Bakheit A.M., Fedorova N.V., Skoromets A.A., Timerbaeva S.L., Bhakta B.B., Coxon L. (2004). The beneficial antispasticity effect of botulinum toxin type A is maintained after repeated treatment cycles. J. Neurol. Neurosurg. Psychiatry.

[24] Catz A., Barkol H., Steinberg F., Ronen J., Bluvshtein V., Keren O. (2007). Repeated botulinum toxin injections can improve mobility in patients with spinal cord lesions. Eur. Medicophys..

[25] Fattal-Valevski A., Domenievitz D., Giladi N., Wientroub S., Hayek S. (2008). Long-term effect of repeated injections of botulinum toxin in children with cerebral palsy: A prospective study. J. Child. Orthop..

[26] Atalay N.Ş., Akkaya N., Aysun Ö., Şahin F. (2012). Hemipleji sonrası gelişen spastisitede botulinum toksin A’nın etkinliği. Anatol. J. Clin. Investig..

[27] Intiso D., Simone V., Bartolo M., Santamato A., Ranieri M., Gatta M.T., Di Rienzo F. (2020). High Dosage of Botulinum Toxin Type A in Adult Subjects with Spasticity Following Acquired Central Nervous System Damage: Where Are We at?. Toxins.

[28] Dressler D., Saberi F.A., Kollewe K., Schrader C. (2015). Safety aspects of incobotulinumtoxinA high-dose therapy. J. Neural Transm..

[29] Bakheit A.M., Severa S., Cosgrove A., Morton R., Roussounis S.H., Doderlein L., Lin J.P. (2001). Safety profile and efficacy of botulinum toxin A (Dysport) in children with muscle spasticity. Dev. Med. Child. Neurol..

